# The lived experience of patients with conflict associated injuries whose wounds are affected by antimicrobial resistant organisms: a qualitative study from northwest Syria

**DOI:** 10.1186/s13031-023-00501-4

**Published:** 2023-01-21

**Authors:** Ahmet Aldbis, Hady Naal, Tarik Kishawi, Rim Wazni, Aula Abbara

**Affiliations:** 1 UOSSM (Union of Medical Care and Relief Organisations), Gaziantep, Turkey; 2 Global Health Institute at the American University of Beirut, Beirut, Lebanon; 3 Department of Infection, Imperial College, Praed Street, London, W2 1NY UK; 4 Syria Public Health Network, London, UK

**Keywords:** Syrian war wounded, Antimicrobial resistance, Conflict, Health system

## Abstract

**Introduction:**

For those with severe conflict-associated wounds which are affected by antimicrobial resistant (AMR) organisms, health systems during protracted conflict are often ill-equipped to respond to their needs. In this study, our aim is to explore the experiences of those with conflict-associated wounds whose wounds have been infected with AMR bacteria and who reside in northwest Syria (NWS). This is with a view to understanding the challenges they face and how the health and humanitarian system can better respond to their needs.

**Methods:**

A qualitative research methodology where in-depth interviews were conducted with patients who are known to have AMR organisms infecting conflict-associated wounds was used. Patients were recruited from Bab Al-Hawa hospital in NWS based on pre-set inclusion criteria. They were invited to participate in remote interviews due to the ongoing COVID-19 pandemic. Interviews were conducted during January and February 2021 and transcribed in Arabic before thematic analysis was undertaken to identify key themes and subthemes.

**Results:**

14 in-depth interviews were conducted of which 12 were with men. The age range was 20–49 years. We categorised the findings into 6 themes: i. those related to the mechanism of injury, ii, the impact of the conflict on health system accessibility, iii. experiences of immediate inpatient management, iv. the experience of outpatient and home management, v. the current impact of the injury on participants, and vi. participant perspectives around improving healthcare access for those with conflict-related wounds affected by AMR organisms. Important findings relate to the quality and capacity for both immediate and longer-term care and the psychosocial and socioeconomic impacts of the injuries which many of the participants continue to grapple with.

**Conclusion:**

This is the first exploration through qualitative research of the experiences of those with conflict-affected wounds which are infected with AMR organisms in NWS. Emerging themes as told by participants can help stakeholders, including policy makers, humanitarian organisations and those involved with health system planning in NWS consider gaps in current and future care needs (including livelihood opportunities) for this vulnerable group.

## Introduction

After over a decade of conflict, Syria’s health system has been decimated, leaving it politicized, fragmented, and ill-equipped to respond to the health needs of its population. For those with chronic injuries, particularly those who are severely disabled or who have complex needs, healthcare access is particularly challenging with up to 70% of health facilities no longer functioning [1]. Northwest Syria (NWS), an area which now shelters around 4.2 million civilians of whom half are internally displaced, has seen ongoing violence and attacks on healthcare, overwhelming the already overburdened health system and impeding healthcare access, including to the most vulnerable[2].

A range of weapons have been used in Syria including conventional weapons, barrel bombs, and chemical weapons resulting in hundreds of thousands of injuries including multiple and complex injuries[2]. The forced displacement of skilled surgeons, physicians, and nurses who can manage such wounds can result in poor wound management including inadequate debridement or surgery, poor wound care, and poor medical management; this can leave wounds susceptible to acute chronic wound infections [1, 3].This, together with the destruction of microbiology laboratories, poor access to infection specialists, weak antimicrobial stewardship, and pre-conflict factors, are drivers of antimicrobial resistance (AMR) in such patients[1] Anecdotal evidence from doctors in Syria suggests that AMR among Syrians is increasing with difficulty to treat infections which do not respond to standard antimicrobials[1]. Results from the few functioning microbiology laboratories also indicate high levels of AMR [4]. Though little data has been published from within Syria, reports from neighbouring countries which host Syrian refugees indicate high levels of AMR in screening, and including around two-thirds of Gram-negative infections in patients injured during the war[1].

Those who are most affected by AMR are likely to be those with deep or chronic, non-healing injuries who may have received recurrent courses of antibiotics (often available over the counter), had frequent visits to healthcare facilities, or those who have had prolonged admissions, repeated surgeries, or poor wound care[1]. An association has also been noted between metalwork and AMR in war-injured Syrian patients in Jordan[5].

Despite the burden of injuries including those with AMR and the impact on patients and their families, there has been no in-depth qualitative exploration of the experiences of patients injured during the Syrian conflict who have conflict associated wounds, affected by AMR bacteria. Additionally, it can be difficult to tease out the effects of AMR as experienced by patients, above the effects of the wounds themselves. Our aim is to explore the experiences and trajectories of patients in NWS with conflict associated wounds affected by AMR bacteria; as such, we placed less emphasis on the exact mechanism of injury than on the result. This is with the aim of understanding the circumstances of their injury, the personal and socioeconomic consequences, their experiences as patients, and any challenges around healthcare access in NWS they may have faced, with a view to understanding the challenges they face and how the health and humanitarian system can better respond to their needs.

## Methods

A qualitative study methodology was adopted using a phenomenology approach through which semi-structured interviews were conducted with patients in NWS who have conflict-associated wounds affected by AMR bacteria. This approach was deemed best suited for the purposes of the present research because of its emphasis on in-depth explorations of the trajectory of patients from the circumstances of wounding, through wound infection to accessing healthcare support and their current situation. Interviews were conducted remotely (due to the COVID-19 pandemic) during January 2021.

### Study setting

The study was conducted in NWS, an area in which around 4.17 million Syrians reside, of whom more than half are internally displaced. This area has faced significant bombardment by the government of Syria and its allies, leaving large numbers of civilians wounded, and the health system in the area devastated. Around 1.4 million of the population in NWS reside in informal tented settlements while others, including those with disabilities, reside in rented accommodation or substandard shelters.

Patients were selected from Bab Al Hawa hospital. This hospital was established by humanitarian organisations and is situated between the Turkish and Syrian borders. It provides inpatient and outpatient care for patients including specialist surgeries (including orthopaedics, neurosurgery, general surgery) for both conflict and non-conflict related injuries. It also offers a basic microbiology service, and it is from the results of this laboratory that patients with AMR bacteria were identified.

### Participants and sampling approach

Participant selection followed a purposive sampling approach because we specifically targeted individuals who were identified by the hospital as having AMR organisms in conflict-associated wounds. Eligibility criteria required participants to be aged 18 years or older, able to consent for themselves, have conflict associated wounds, and have confirmed AMR organisms causing infections in their wounds (i.e. not only colonising).

### Procedures

Upon receiving ethical approval from the American University of Beirut, and permission from Bab al Hawa hospital to proceed with this study, the data entry specialist at Bab Al-Hawa Hospital contacted patients who had been identified as fulfilling the inclusion criteria in order to acquire preliminary approval to conduct the interviews. Following this initial permission, the main interviewer (AD) contacted these patients and shared the Participant Information Sheet (PIS) and the informed consent forms in the written form in Arabic where possible. When this was not possible (e.g. due to illiteracy or logistical constrains such as lockdowns or security concerns), AD provided detailed verbal information of the study and process.

AD gave potential participants 48 h to read the information provided and recontacted them after to answer any questions and arrange an interview if they consented. Upon receiving consent from patients, interviews were arranged to be conducted in Arabic, and remotely over the phone to abide by data collection policies set forth during the COVID-19 pandemic. In specific, WhatsApp phone application was used, as most patients had access to this application but not to others. All interviews were recorded and transcribed verbatim in their original language, taking into account the confidentiality of data and the privacy of participants, and their approval to have the interview audio recorded. Data were collected between 20th January 2021 and 28th February 2021, and they lasted for 30 min except for two (both conducted with women) which lasted for 60 min.

To protect the anonymity of participants, verbal informed consent was obtained from all participants prior to data collection. All data were anonymised with participants assigned a participant code. Only the interviewer (AD) who also performed the analysis had access to the data and it was stored on a single password protected drive. AD completed the transcriptions in Arabic and was the only study member to have access to them. Participants were informed that all data will be destroyed 3 years after the research is completed and published. Participants were aware that they could withdraw their participation 24 h before data analysis began and all information relevant to the participant would be deleted.

### Data collection tool

For the purposes of this study, the research team developed a semi-structured interview form to guide the data collection process. The interview questions were all open-ended, originally developed in English but translated to Arabic, and the addressed the following categories: 1. Timing and mechanism of initial injury 2. Experiences of accessing healthcare including medical or surgical treatments and antimicrobials 3. Experiences of interacting with the local health system as an inpatient and outpatient. The questionnaire is available in appendix (annex1).

### Data analysis

After transcribing the recorded interviews verbatim in Arabic, they were cross-checked to ensure accuracy. It should be noted that the research team has adequate understanding and familiarity with the cultural landscape of the target population given that some of the members are Syrians or have worked with Syrians previously. The data was then analysed using qualitative content analysis following an inductive approach, whereby codes were first identified, leading up to the development of categories and themes of analysis. A thematic framework was then created, whereby the topics were indexed, sorted, given symbols, and then linked to one another. Throughout the coding process, the research team met regularly to discuss coding and data analysis, review findings, and agree on emerging themes and codes.

### Ethical considerations

Ethical approval for this study was granted by the Institutional Review Board (IRB) at the American University of Beirut (AUB). SBS-2020-0299. It was not possible to obtain local ethical approval in NWS due to the absence of a functioning ethical review board however permissions from Idlib Health Directorate from where the patients were selected and the manager of Bab Al Hawa were obtained.

Due to the nature of the participants and potential concerns about their wellbeing, should any participant show signs of distress, the interview would be terminated and the number to local, accessible mental health services would be provided.

## Results

### Participants

Of 25 potential participants whose isolates were screened (2 of whom were women), 16 had AMR organisms and were approached to participate; 14 gave their consent to participate, and based on our analysis, this number was enough to reach data saturation. The participants were almost all men (12 of the 14), their age range was 20–49 years, and most resided in the governorate of Idlib in NWS. The participant characteristics are detailed in Table 1.

**Table 1:** This table lists the participants, their age range, location, type and mode of injury

	n (%)
*Age*	
20–29	6 (43)
30–39	4 (29)
40–49	4 (29)
*Gender*	
Man	12 (86)
Woman	2 (14)
*Governorate*	
Idlib	12 (86)
Aleppo	2 (14)
*Mechanism of Injury*	
Light weapon	1 (7)
Missile bombing	2 (14)
Airplain bombing	9 (64)
Explosive device	1 (7)
Mortar shell fragment	1 (7)
*Resulting injury*	
Gunshot wound to the left hip	1 (7)
Abdominal and back wounds with damage to the liver, spleen and intestines	1 (7)
Peneteratimg wound to the shoulder and chest with physical and nerve loss	1 (7)
Shrapnel injury which led to the amputation of one leg	1 (7)
Head injury and shrapnel injury to the trunk	1 (7)
Shrapnel injury to the back and spinal cord damage	1 (7)
Injury to both legs with physical and neurological disaibility	1 (7)
Large head wound and irreversible damage to the sclera	1 (7)
Shrapnel injury to back, legs and abdomen	1 (7)
Shrapnel injury to the abdomen with splenic, renal and intestinal damage	1 (7)
Shrapnel injury to the knee	1 (7)
Shrapnel injury to the abdomen, upper limbs, and head	1 (7)
Shrapnel injuries to the abdomen and legs with significant loss of function and nerve damage	1 (7)
Shrapnel injury to the thigh with significant loss of function and nerve damage	1 (7)

As reported by patients

**Fig. 1:**
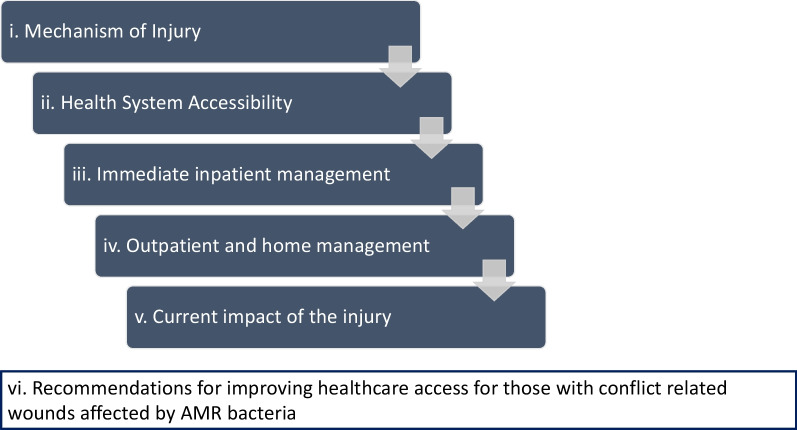
The main emerging themes are summarised in this figure

We categorised the key findings into six main themes, each of which had sub-themes within them. These are summarised in Fig. 1. They were prioritised according to the frequency and emphasis of mention by the participants.

#### Mechanism of injury

Most of the participants described injuries related to shrapnel (9 of 14) from a range of weaponry including explosive devices, aircraft bombings, or barrel bombs with one describing injury from a light weapon and others from missiles. Five reported injuries resulting from barrel bombs, and four from vacuum missiles. Participants reported a range of injuries as a result, including severe ones which required multiple surgeries and antibiotics courses; such factors could affect the development of AMR. Reported attacks occurred in marketplaces, on roads while travelling to escape shelling and of residential homes. One participant reported the mechanism of his injury during Ramadan:

“During Ramadan, an hour before the call to prayer, I went to the only market in the besieged town (Dareya) to buy some food for my children and wife. At that moment, the market was targeted with a barrel bomb causing buildings to collapse. I was injured but remained conscious and lay under the rubble for 3 hours.”

Participants noted that delays and severity of injury increased their chances of infected wounds as well as risks of contamination from the environment. Crush injuries from barrel bombs were noted to cause among the most severe injuries which were difficult for immediate responders or surgeons to clean or close.

A woman participant in her early twenties described how a vacuum missile caused her injury. She reports:

“After my city was bombarded, my family and I were forced to leave in our cars to avoid death. We were directly targeted by a vacuum missile on the road, which resulted in a massive shrapnel injury to my shoulder that penetrated my chest. The road was almost deserted but once farmers saw our cars burning, they hurriedly transferred us to a nearby hospital in Maarat al-Numan.”

#### Impact of the conflict on health system accessibility

Most participants noted the impact of (at that time) almost a decade of bombardment on the health system in NWS and its ability to manage the acute injuries which they faced. They noted that some emergency providers had inadequate training, lacked resources and relevant skills which could worsen the immediate injury. Often, they would not use sterile equipment on the open wounds. They also described delays in emergency services reaching them. One participant describes his journey to hospital:

“After I was injured by shrapnel in the knee as a consequence of an artillery bomb while I was in the farm, and as the bombing continued, I bled for 3 h until ambulances were able to reach me.”

Another noted that “Because of the bombing, no one could reach me until I got out [from under the rubble] on my own. I walked bleeding for a distance of more than 500 m until I found a person driving a motorcycle who took me to the hospital.”

Some noted that even while being transported to hospital or a medical point, they felt unsafe due to ongoing targeting by air missiles or snipers, particularly in areas close to the frontline or in besieged areas. One participant noted:

“While I was being taken by car from eastern Aleppo to Bab al-Hawa Hospital at night, the road was very dangerous. We felt that the aeroplane is always above us and we were always afraid. It took more than 8 hours till we arrived at the hospital.”

#### Experiences of immediate inpatient management

Participants reported that due to the inexperience of some the emergency providers, they were often taken to the closest rather than the most appropriate health facility which could respond to the participants’ immediate needs. This was due to the emergency providers being unable to accurate assess the severity of the injury and the required services. One participant reported:

“The paramedics took me to Ma’arrat al-Numan hospital, which is close to where I was injured, but my injury was severe. The hospital’s capabilities were insufficient to deal with my critical condition.”

Another reported:

“When I arrived at the hospital, the medical staff offered me basic first aid procedures only as they were not able to do anything more advanced.”

This was the case, even in Bab Al-Hawa Hospital which was one of the best equipped hospitals which could offer emergency and surgical care in the area. One noted:

“The hospitals where I was treated offered me everything they could, but my injury was so extensive and intricate that neither the supplies nor the drugs I required were available. As a result, after eight days in Bab Al-Hawa Hospital, I was sent to Turkey for further treatment.”

Other subthemes included the absence of adequate diagnostics and specialists; the lack of sufficient specialists was noted by a number of participants who reported that a single doctor without specialist training may need to respond to all specialist surgical emergencies leaving them concerned about post-operative complications. Other participants also commented on the inadequate quality of medical care immediately after surgery with early discharges, sometimes due to shelling of facilities and other times due to insufficient staff or resources. Services in NWS are often provided by humanitarian organisations who face difficulties in funding, shortages of healthcare staff and continuous bombing. Participants were concerned as to whether this contributed to wound infections and the potential for AMR to develop if repeated antibiotic courses were needed.

#### Experience of outpatient and home management

Given the diversity of injuries and resulting disability, as well as the home situation, including those related to gender and religious factors as well as who was available to provide care for them, the experiences of participants after discharge were quite different.

Ideally, those with severe injuries, particularly if bed-bound or had ongoing open wounds which required specialist care, should have access to an appropriate bed, an air mattress, and adjustments for toileting as well as required supplies to keep wounds clean including gauze, cotton, sterilizers, or medicines. However, given the poor living situation, the long duration of treatment and associated high costs and the costs of equipment or needed adjustments, patients were often unable to access these. For Internally Displaced People (IDPs) the housing situation prevented an appropriate home environment. One participant reports:

“I live in a single tent with six family members; there are no hygienic conditions, no fuel for heating, and I have many injuries.”

Even for those not forcibly displaced, living conditions could be challenging. Another participant reports:

“Our house was partially destroyed due to the previous bombing, but we have no other place to live, and we were unable to secure a hospital bed, so I used the normal sleeping mattress during my home management which caused me harm.”

Others, particularly in besieged areas, were forced to live underground in basements or in caves where ventilation was inadequate and where there was no exposure to sunlight. This potentially contributed to worsening of wounds or injuries and a slower recovery. A participant reported:

“My family and I lived in a besieged area, and our house lacked everything, including running water, power, and even air and sunlight. In addition, because of the massive displacements, during which the displaced cannot even secure a tent or a room, people were forced to live cooperatively and in overcrowded conditions.”

Participants noted other shortfalls in outpatient management which they felt could have contributed poorer surgical or wound outcomes or to the development of AMR. The costs of medications and consumables such as dressings/ antiseptics could be prohibitive, particularly if the participant was the main breadwinner for the family and could not work. Even if they could afford certain supplies or antibiotics, they may not be available or of sufficient quality which some participants felt could worsen their response or lead to AMR and failure of antibiotics. For the women participants, they noted that a women healthcare worker may not be available in their area; this was noted to be important given local traditions and religious beliefs. One of the two women participants interviewed noted:

“Unfortunately for me, there were no women medical workers near my residence, which caused some humiliation during the wound dressing and debridement.”

For AMR, this could mean that wounds are inadequately treated which could result in ongoing infection requiring further courses of antibiotics.

#### Current impact of the injury on participants

Almost all participants reported current effects of their injury on their physical and mental health, as well as on their perceived role in society. Some reported that they felt they were a burden on their families, could not work to support their families, or participate fully in social activities. One participant noted:

“Although my injury was about two years old, I am still in the treatment phase, and I have a permanent disability. I feel that society looks at me with an uncomfortable look and that I am not human; this leaves a great psychological impact on me." Another says: “Before the injury, I was able to drive a car and a tractor, and support my family financially, but now I am helpless, and I am seeking help from others.”

#### Improving healthcare access for those with conflict related injuries affected by AMR

At the end of the interview, participants were asked what their views were on improving the situation for those with conflict-associated injuries which are affected by AMR would be. Answers included those which relate to availability and quality of treatment (taking into account both physical and mental health,) those relating to rehabilitation, and those which related to the place of those with conflict-associated injuries in society. A participant noted:

“I wish there were someone who could monitor my medical state and therapy. I am not the only one who requires assistance and other injured people are in a worse predicament. There must be someone in charge of their care and the monitoring of their medication or medical intervention needs.” She also noted, “My injuries and their psychological impact, as well as my involvement in humanitarian efforts to help others, keep me from thinking about my damage and its implications. However, now I feel worthless in society; no one cares if I live or die, and no one would even hear about me.” She added, "This interview allowed me to communicate freely about my agony and suffering, and for the first time, I believe that there are people who care about us as wounded people when we had given up hope.”

Another participant focused on the quality of hospital care, its costs, and the need for longer term financial aid. He noted, “We as wounded are harmed by a shortage of free hospitals, and there are private hospitals that charge exorbitant fees. I would like hospitals to follow up on the wounded better and for free till they heal. Organisations also need to consider supporting the injured with work and a chance for each injured person to work according to his injury to secure an income, even at a basic level, to support a decent life.”

Another participant shared a similar sentiment stating, “I wish there is a group of specialized staff to take care of the wounded and provide for their needs. This could include supporting with employment opportunities or support for studying or training.”

One participant ended with the hope that “the war will come to an end and that people will return to their homes and lands, live in safety, and take care of the wounded and their needs.”

## Discussion

Through this qualitative study we aimed to highlight the experiences of those who had conflict-associated wounds which had been affected by AMR bacteria in NWS. By following their trajectory from the time and circumstances of wounding through their treatment and interactions with the healthcare system to the current challenges they face, we hope to draw attention to the situation of this socially and economically marginalised group. For clarity, and as mentioned above, we categorised the findings into 6 themes: i. those related to the mechanism of injury, ii, the impact of the conflict on health system accessibility, iii. experiences of immediate inpatient treatment, iv. the experience of outpatient and home treatment, v. the current impact of the injury on participants and vi. recommendations for improving healthcare access for those with conflict related wounds affected by AMR bacteria. The participants chosen had wounds affected by AMR, since our goal was to explore their experiences and perspectives. Given the methodology, we did not aim to compare to the experiences of those unaffected by AMR.

In general, the most important findings relate to the quality and capacity for both immediate and longer-term care in a devastated health system and the psychosocial and socioeconomic impacts of the injuries, which many of the participants continue to grapple with. The impact of AMR among participants is hard to differentiate from among the experiences of those with comparable conflict-related wounds without AMR, however it is likely that the rate of healing, need for prolonged and more expensive antibiotics, and poorer wound outcomes influenced the experiences of patients in whom AMR bacteria affected their wounds [1, 6]. In addition, when exploring the circumstances of the wound and the impact of the conflict-weakened health system in its ability to respond to those with conflict-associated wounds, there are potential factors which contribute either directly or indirectly to the emergence of AMR organisms. Beyond this, given more prolonged interactions with the health system, the socioeconomic consequences may be greater for those affected by AMR. Future larger scale quantitative research should further explore and quantify this.

In this qualitative study with a relatively small cohort and without an AMR unaffected comparison group, the associations between the type of weapon and the development of AMR cannot be assessed. However, the presence of heavy metals in armed conflicts as a potentiator of AMR through the selection for antibiotics and heavy metal co-resistance mechanisms is increasingly explored [7]. With regards to the development of AMR in such settings, a review by Sahli et al. [8] of conflict related wound infections in the Middle East noted certain risk factors for AMR in such wounds including excess antimicrobial prescriptions, inadequate prophylaxis at the time of injury, the presence of metal work as well as health system related factors. Additionally, the presence of more severe or complex wounds may lead to the development of AMR. A study from an MSF facility in Raqqa noted that they received a second, larger and more complex array of blast wounded patients when the population returned to the area which has IEDs (improvised explosive devises) and ERWs (explosive remnants of war) [9].The severity of such wounds in a health system which is unable to manage them effectively, can lead to prolonged infections, recurrent courses of antibiotics and the development of AMR.

### Impact of a devastated health system

The health system in NWS has faced significant and ongoing destruction of healthcare including health facilities and ambulances which have adverse effects on access to healthcare [10–12]. For acutely injured patients, this often means delays in emergency services reaching them and, due to the targeting and forced displacement of healthcare workers [13] alongside inadequate training, poor quality of emergency care and transport which can lead to longer term harm. Some participants noted that poor pre-hospital care or transfer to a facility without the required expertise or resources could worsen their condition. Of concern were risks associated with ongoing bombardment or targeting during their transfer to hospital which could take prolonged periods due to check-points and unsafe road conditions [14]. There was little information on pre-hospital care in Syria though Wong et al. [10] noted in their analysis of attacks on ambulances that the intentional and repeated targeting of ambulances, risks of ‘double-tap’ attacks can lead to delays to care with potential acute and long-term consequences. With regards to risks of AMR, antibiotic prophylaxis is recommended for all severe, acute injuries with open wounds however in NWS, given the poor state of emergency and pre-hospital services, this is unlikely to be provided routinely and even when provided, it is unlikely that patients will receive the right antibiotics at the right doses at the right time. In addition, delays to administration of antibiotics, first debridement or an incorrect intervention e.g., placing internal fixation in an open wound may increase the risks of infection and the subsequent development of AMR [8] Information on the use of prophylactic antibiotics in this cohort was not available given inadequate patient records and patients would be unaware of what they received at the time of injury or surgery.

Delays to immediate, appropriate management of wounds can contribute to AMR through inadequate early debridement or wound management. Such interruptions may be due to attacks against immediate responders or patients and roadblocks or checkpoints which cause delay in arrival or transfer 15 [18]. A 2016 review by Safeguarding Health in Conflict noted that such attacks occurred in 23 countries including on pre-hospital care [14] in countries such as Afghanistan, Democratic Republic of the Congo, Iraq, Niger, Mali and Yemen [14, 15]. ﻿In the Occupied Palestinian Territories, there were 416 instances of violence or interferences on ambulances owned by the Palestinian Red Crescent which resulted in injuries to volunteers, interruption of aid to patients and damage to 108 ambulances in that year [14]. Such attacks may lead to delays to wound management, inadequate prophylaxis which could contribute to AMR. Participants noted this, commenting on health system factors and inadequacy of emergency services or appropriateness of immediate care.

First responders may take patients to the nearest facility rather than the most appropriate given insecurity or a lack of familiarity with what patients may need versus what the health facilities can provide. Participants reported that hospitals were often functioning beyond capacity, particularly in areas which were besieged such as Eastern Ghouta, eastern Aleppo or parts of Homs; this contributed to the scarcity of antibiotics, other required medicines as well as those with the expertise to manage the injuries [11]. Overcrowding and poor infection prevention measures in health facilities can also lead to nosocomial transmission of AMR bacteria [8] as seen in conflicts in the Middle East; this can lead healthcare facilities to contribute to the AMR burden in these settings.

Poor outpatient wound care and access to required healthcare may also increase the risk of developing wound infections which could become colonised or infected with AMR bacteria; this is of particular concern in those who have metalwork in place as infections can be harder to cure with antibiotics alone due to biofilm formation [8]. The presence of AMR can also increase associated costs for patients due to increased costs of antibiotics as well potentially more protracted wound infections. Despite the frequency with which patients note the socio-economic impact of AMR in their wounds, this topic remains understudied. A review by Kobeissi et al. [16] noted a paucity of literature on the socioeconomic burden of AMR among populations in conflict-settings, identifying only 8 studies with most studies not having assessed the socioeconomic burden as a primary aim; they were also small studies with the limited statistical power.

The forced exodus of healthcare workers as well as inadequate training can also contribute to poor wound care, inadequate surgical interventions and poor medical care [13]; the latter may include overprescribing or incorrect prescribing of antimicrobials which can lead to selective pressures resulting in increased AMR [1]. For clinicians, it is essential to differentiate between colonising bacteria versus those which are causing infection to ensure that there judicious antimicrobial prescribing [17]. This is hampered by the poor microbiology support in many areas affected by conflict which can lead to inaccurate antibiotic susceptibility data which can contribute to the problem. A review of bone cultures from war-wounded civilians in MSF’s project between August 2006 and January 2016 in Jordan found that 55% of 1353 bone samples had AMR; patient had originated from Iraq, Yemen, Syria and Gaza [18]. Importantly, they noted that in some patients, osteomyelitis was not suspected, and these patients would otherwise have had internal fixation if the bone cultures had not grown bacteria. In Syria, some efforts to strengthen services through tele-microbiology have been trialled but have yet to be introduces sustainably, particularly where ongoing attacks on health facilities including laboratories occur [19].

### Socioeconomic impacts

Socioeconomic factors and poor living conditions were noted by almost all participants, relating to access to employment as well as inability to afford essential treatments or services for their clinical needs. Resource and transport constraints also affected their ability to travel to health facilities for medical care or physiotherapy. Poverty in Syria has reached more than 80% of the population with high rates of unemployment, particularly after the COVID-19 pandemic. For some, who may be the head of the household, unemployment could also push their families further into poverty leading them to need to either reliable on handouts or charities and to make difficult decisions to prioritise food and shelter over their own medical needs. These impacts among patients with conflict-associated wounds remain understudied however Dewachi’s ethnographic exploration of such wounds explores the as a ‘social wound’ with intersections in social experiences for those who also carry physical wounds [20]. In NWS where this study was conducted, around 1.4 million IDPs reside in tented settlements with others living in inadequate shelters. For participants who had significant disabilities, they lacked the ongoing physiotherapy, equipment and adjustments to shelter that would have allowed them a better quality of life. The lack of adaptations and costs of transport also affected their ability to keep outpatient appointments. Such factors are unlikely to be related directly to AMR in itself but to the severity of their injury and resulting disability.

### Addressing the needs of those with conflict wounds affected by AMR

The complex needs of those with life-altering conflict related wounds, particularly those with AMR require expertise which takes a holistic, patient-centred approach to ensure effective management. Levesque et al. [12] conceptual framework of access to healthcare provides a useful way in which to explore the health system needs of those with AMR affected conflict wounds. Such patients require a multi-disciplinary approach which considers both the medical needs as well the psychosocial needs of such patients and their abilities to perceive, seek, reach, pay for or engage with services [12].

Prior to Syria’s conflict, surgeons and physicians lacked expertise in the care of war related injuries and had to develop such skills during the conflict. With regards to AMR, significant challenges remain with regards to expertise, laboratory back up as well as the availability of required medications to effectively manage AMR affected wounds, particularly for those which have metalwork in place [1]. Though some efforts to address resources and expertise gaps have been made, the ongoing conflict, high population needs and challenges relating to cross-border aid have impeded efforts to strengthen the health system in the area. It is essential that humanitarian organisations, policy makers and funders take into account the needs of patients with conflict injuries and strengthen resources for investigation and management of AMR.

Participants stressed the need for both physical and psychosocial support but all emphasised that opportunities for employment so that they can support themselves and their families were essential. In NWS, given the inadequacy of the humanitarian response compared to the increasing needs of the population, the needs of those with disabilities are often overlooked. Initiatives in areas under government control are also sparse but some promising projects which seek to support those with disabilities with free physiotherapy, artificial limbs and other aids has been in Hanano Primary Health Care centre in Aleppo since 2018; it provides services to 3500 community members per month [21]. However, without active policies which support inclusion and reduce discrimination faced by those who have significant disabilities as a result of their injuries, livelihood and economic opportunities will be unavailable to them both immediately or in the early reconstruction phase. However, with high unemployment across Syria and limited social policies to support marginalised populations, those with disabilities are often overlooked in health system and humanitarian planning [22].

Policies focused on long-term inclusion are essential due to permanent nature of many of the disabilities among this population though the interplay between disability and AMR (including socioeconomic and clinical outcomes) is poorly characterised in the literature, even outside of Syria. A December 2020 HNAP (Humanitarian Needs Assessment Program) factsheet on disability in Syria estimates that 30% of those over the age of 12 years have a disability of which around 18% relate to mobility with socio-economic and psychosocial impacts [23] It is also estimated that 1.4% of all years lived with disability relate to violence and conflict with rates of disability close related to violence and conflict [24]. Data from Vietnam suggests a positive association with bombing on disability more than 30 years after the conflict ended, suggesting a long-term impact of conflict on individuals as well as the health system [24].

The particular needs of women who have conflict associated wounds affected by AMR are important to understand. However, only two of the participants were women (and of the 25 initially screened) and both agreed to participate. Of note, their interviews lasted twice as long on average as the interviews with men. The disparities in numbers of women may related to the greater number of men involved in combat compared to women or to the ability of injured women to access specialist facilities due to increased reliance on other family members. Gender related factors were important in terms of access to services, availability of women healthcare staff and sociocultural impacts and suggest an intersectionality of vulnerabilities, additional to that of men participants. One of the women participants also highlighted the religious and gendered needs which affected her care and caused her distress at the time of her wound changes due to a lack of women medical workers where she resided. Based on a sample of two men, we are unable to extrapolate more from this however further work which explores the gendered elements of this cohort is needed.

## Strengths and limitations

Though all patients identified had AMR bacteria in their conflict associated wounds, it is hard to tease out whether and how the experiences of those with AMR differ compared to those without AMR. It is likely that other factors, for example the mechanism, timing, and extent of initial injury and subsequent disability, family, social and economic support as well as experiences of interacting with healthcare have a more prominent role in influencing the experiences of those with conflict associated wounds. The age range of participants is limited at 20–45 and only 2 of the 14 participants were women; as such, their experiences are likely to be different for those outside of this age group in terms of comorbidities, responsibilities, or social support and gender-related challenges which may not have been fully explored in this study. Despite this, the range of participants in terms of range of experiences, geographical area of initial injury, have some diversity though the current location of all participants is Idlib or Aleppo governorates in areas under opposition control in NWS, as this is where we sampled from. As such, in terms of experiences with the healthcare system, it may not be generalisable to other parts of Syria given differences in the subnational health systems across Syria, knowing that generalizability was beyond the scope of this study; the focus was on drawing an in-depth understanding of the experiences of the target group. Many participants stated that this was an important opportunity for them to have their voices heard with the hope that consideration for their needs can be factored into current and future health system planning.

## Conclusion

This is the first qualitative study which presents the experiences of those with conflict-associated wounds affected by AMR bacteria in NWS. It brings to light various factors which may adversely or positively affect such patients as well as gaps in the current and future provisions for their care. It also highlights the individual experiences of those whose lives have been permanently altered by injuries which have resulted from attacks the Syrian government and its allies. We hope this work is of interest to policy makers, humanitarian organisations and those involved in health system planning who can draw on the experiences of participants to ensure that inclusive practices which consider their health needs are developed. As the health system in NWS is increasing underfunded and.

The particular needs of patients with protracted conflict related injuries, particular those affected by AMR (who may have worse outcomes including non-healing wounds) need to be considered. This work acts as a starting point for future exploration of the experiences of those with conflict associated wounds, disabilities, and AMR as a result of the ongoing conflict in Syria with a view to affecting health and social policy changes to meet their needs.

## Data Availability

Given the sensitive nature of some of the interviews, we have not made the raw data publicly available. Further information can be obtained from the corresponding author.

## Appendix

### Question Guide

A qualitative study of experiences Syrian war wounded whose injuries are affected by antimicrobial resistance.

### Personal information

Participant code:

Age:       50 <        > 50

Marital status:   Married            Single

Number of your family members: 2 <           > 2

Educational level: Preparatory school    secondary school    University study

Occupation:         work       not work

Place of residence: Camp      tent      house      other.

Chronic diseases. Diabetes     asthma       Heart disease      Hepatitis      other.

During the interview, you will not be requested to identify any third party by their name.

### Injury occurrence phase

- Tell me about how the injury occurred and the circumstances around it?
- Tell me in detail about the first aid which was provided to you before you was transferred to hospital?
- Detail the steps that were taken to transfer you to the hospital?

### Injury Treatment phase

- Describe what happened in the hospital when you arrived?
- What treatment did you receive in hospital, and how long did it last?
- Tell me about your treatment conditions after you finished it in the hospital?
- Describe your condition in the current situation after you have finished all the stages of treatment?

### Challenges and difficulties during the treatment phase

- Tell me in detail about the challenges and failure points that you think have negatively affected your injury from the time you got it until recovery?

### Challenges and difficulties after treatment phase

- Describe to me your current circumstances and the challenges you face as a result of the injury and its impact on your life?

## Abbreviations

AMR: Antimicrobial resistance
HNAP: Humanitarian Needs Assessment Program
IDPs: Internally displaced persons
IEDs: Improvised explosive devises
NWS: Northwest Syria
WHO: World Health Organization
